# Fluorescence brightness and photostability of individual copper (I) oxide nanocubes

**DOI:** 10.1038/s41598-017-17295-0

**Published:** 2017-12-04

**Authors:** Nafisa Zohora, Ahmad Esmaielzadeh Kandjani, Antony Orth, Hannah M. Brown, Mark R. Hutchinson, Brant C. Gibson

**Affiliations:** 1 ARC Centre of Excellence for Nanoscale BioPhotonics, School of Science, RMIT University, Melbourne, VIC 3001 Australia; 2 Centre for Advanced Materials and Industrial Chemistry, School of Science, RMIT University, Melbourne, VIC 3001 Australia; 3 ARC Centre of Excellence for Nanoscale BioPhotonics, Robinson Research Institute, Adelaide Medical School,The University of Adelaide, Adelaide, SA 5005 Australia; 4 ARC Centre of Excellence for Nanoscale BioPhotonics, Adelaide Medical School, University of Adelaide, Adelaide, SA 5005 Australia

## Abstract

Conventional organic fluorophores lose their ability to fluoresce after repeated exposure to excitation light due to photobleaching. Therefore, research into emerging bright and photostable nanomaterials has become of great interest for a range of applications such as bio-imaging and tracking. Among these emerging fluorophores, metal oxide-based nanomaterials have attracted significant attention as a potential multifunctional material with photocatalytic and angeogenisis abilities in addition to fluorescnce applications. However, most of these applications are highly dependent on size, morphology, and chemo-physical properties of individual particles. In this manuscript, we present a method to study the intrinsic optical characteristics of individual copper (I) oxide (Cu_2_O) nanocubes. When excited at 520 nm using only 11 µW excitation power (1.7 W/cm2), individual nanocubes were observed to emit light with peak wavelengths ~760 nm which is conveniently within the near-infrared 1 (NIR1) biological window where tissue autofluorescence is minimal. Bright and photostable fluorescence was observed with intensities up to 487 K counts/s under constant illumination for at least 2 minutes with a brightness approximately four times higher than the autofluorescence from a fixed cumulus-oocyte complex. With near-IR emission, high fluorescence brightness, and outstanding photostability, Cu_2_O nanocubes are attractive candidates for long-term fluorescent bioimaging applications.

## Introduction

Copper (I) oxide (Cu_2_O) is a p-type semiconductor material with a direct bandgap 2.17 eV in bulk form^1–5^. This semiconducting material has attracted much attention due to its exceptional properties which are possible in nano-sized particles. Various morphologies are possible such as nanocubes, nanospheres, nanorods and nanooctahedrons, as reported in the literature, synthesised via simple methods and low preparation costs^1–12^. These approaches make this material suitable for scalable manufacturing and provide a competitive edge as an oxide semiconducting material. Copper (I) oxide shows a high absorption coefficient at around 438 nm in bulk and ensembles, has more than 10% energy conversion efficiency and a quantum yield of 6.6 × 10^−2^% when excited at 360 nm and emission at 493 nm^13^ which makes this material a promising candidate for various photoelectronic applications, such as photovoltaic cells and photo-capacitors^13,14^. Other areas of interest which explore the use of Cu_2_O nanoparticles are in the fields of biological imaging^15^ and photocatalysis^3,16^. As an example, Qi *et al*. have used Cu_2_O nanoparticles for light scattering imaging of living cells and as a probe for conformation of proteins^15^, where they report changes in circular dichroism of specific proteins such as prion (PrPC) and bovine serum albumin (BSA) due to the introduction of Cu_2_O nanoparticles. It has been shown that the optical and electrical properties of Cu_2_O semiconductors are highly dependent on their morphology and the growth of the crystal facets of Cu_2_O nanoparticles^12,17^. Among the various morphologies of nano- copper (I) oxides, nanocubic morphologies have attracted great attention due to their well-defined cubic structure and shape homogeneity. Ensembles of Cu_2_O nanoparticles have also been shown to have an intraband photoemission due to oxygen vacancies which give rise to 750 nm emission with 532 nm excitation^18^. The emission at 750 nm makes this nanoparticle a good candidate for bioimaging applications as the emission lies within the NIR1 biological window^19^. However, up until now, the optical fluorescent properties of individual, isolated Cu_2_O nanoparticles have not been studied.

The emerging area of biophotonics requires the development of intrinsically bright and photostable luminescent nanoprobes. Our approach in this paper is to explore the optical properties of individual and isolated cubic Cu_2_O nanoparticles, which are monodisperse in size. Previous characterisation and analytical studies that have been reported in the literature for Cu_2_O nanoparticles have focused on ensemble colloid solutions or bulk materials that are widely dispersed in size and morphology, resulting in cumulative measurements^20,21^. Collecting bright and stable emission from fluorescent organic dyes or nanoparticles using low laser excitation is essential when these fluorophores are used for biological imaging. Biological samples are sophisticated and highly responsive to laser irradiation as high laser power coagulates proteins of tissues, thus it can destroy a sample^22^. Hence, it is desirable for a fluorescent nanoparticle to exhibit bright emission, above that of any surrounding background fluorescence, with minimal optical excitation. In this research, we have examined individual Cu_2_O nanocubes using silicon wafers which have been milled using a focused ion beam to create registration markers^23^. The marked substrates are visible in both a confocal microscope and scanning electron microscope (SEM) and enable the characterisation of isolated nanocubes without interaction from adjacent particles. We now present the first study of the optical fluorescent properties of individual Cu_2_O nanocubes, compare their performance against existing commercially available fluorescent materials, focusing on the intrinsic brightness and photostability of the material for bioimaging applications.

## Experimental

### Chemicals for Cu_2_O synthesis

Copper (II) sulphate (CuSO_4_), Sodium dodecyl sulphate (C_12_H_25_NaO_4_S), (+)-Sodium L- ascorbate (C_6_H_7_NaO_6_) and Sodium hydroxide (NaOH) were used in the synthesis of Cu2O nanocubes. All chemicals were obtained from Sigma-Aldrich and used as received. The water used was double distilled de-ionized Milli-Q water 18.2 MΩ.cm.

### Chemicals used for cumulus-oocyte complex preparation

αMEM supplemented with bovine serum albumin (BSA; ICPbio, Glenfield, New Zealand), Recombinant human follicle-stimulating hormone (50 mIU/ml; Organon, Oss, The Netherlands), equine chorionic gonadotropin (eCG; Folligon, Intervet, Boxmeer, The Netherlands).

### Cu_2_O nanoparticle synthesis

A seed-mediated growth method^1^ was used to synthesise Cu_2_O nanocubes which were tailored to increase the yield of Cu_2_O nanocubes compared to the in the published synthesis process. A solution containing 1 mM of CuSO_4_ and 33 mM Sodium dodecyl sulphate (SDS) was prepared and 30 ml of the prepared mixture was transferred to a round bottle flask (bottle A) followed by the addition of 750 µL of 0.2 M (+)-Sodium L-ascorbate. The solution was vigorously shaken for 5 seconds followed by the addition of 1 M NaOH and shaken another 5 seconds. Then, 20 mL of solution from bottle A was transferred to another round bottle flask (bottle B) with 180 ml of the starting solution and kept in constant shaking for 10 seconds. Then, 5 mL of 0.2 M (+)-Sodium L- ascorbate was added to bottle B and shaken for 5 seconds. Afterwards, 10 mL of 1 M NaOH was added to bottle B and then shaken for another 5 seconds. Sample B was kept standing for one hour. The synthesised Cu_2_O nanocubes were centrifuged at 5000 rpm and washed three times for ten minutes each and redispersed in 10 ml ethanol

### Characterization

The morphological studies of synthesised Cu_2_O nanocubes were carried out with FEI Verios 460 L scanning electron microscope using 10 kV and 0.8 nA. The structural characteristics of the synthesised materials were studied using Bruker D8 Discover microdiffraction system which has general area detector diffraction system and the Cu-Kα radiation source. The oxidation state studies of the prepared samples were studied using Thermo K-Alpha instrument at a pressure better than ~10^–8^ Torr. The core binding energies of the elements were aligned at 285 eV for adventitious C1s core level energy. Si substrates were marked using focused ion beam milling with a FEI Scios FIB-SEM. Each marked area on the silicon has a size of 286 µm × 286 µm with an etched depth of 1 μm. A beam current 3 nA at 30 KV was used for 516 seconds with tilt 52° to mill each substrate. Fluorescence confocal images were taken using a 6 ps pulsed Fianium SuperChrome laser source, at a repetition rate of 40 MHz, with a centre wavelength of 520 nm and a full width at half maximum (FWHM) of 10 nm. The imaging was performed using a 532 nm dichroic mirror, 532 nm long pass filter, 532 nm short pass filter and a 100 × 0.9 NA objective lens.

### Tracking individual particles

Individual Cu_2_O nanocubes were studied using a marked silicon substrate^23^ which was milled with focused ion beam (FIB). The marked Si substrate was drop cast with one drop of the sample. A low magnification SEM image of the deposited, marked substrate was taken to locate regions of isolated particles. Afterwards, the individual particles have been numbered (P1 to P19) and then optical data have been collected the marked individual and isolated Cu_2_O particles.

### Cumulus-oocyte complex (COC) sample preparation

All animal work was approved by the University of Adelaide Animal Ethics Committee. Female mice were administered 5 IU equine chorionic gonadotropin (i.p.) (eCG; Folligon, Intervet, Boxmeer, The Netherlands). 46 hours post-eCG injection, ovaries were collected and COCs liberated from antral follicles. COCs were then placed in maturation medium and matured for 16 hours in a volume of 50 μl medium/COC at 37 °C under paraffin oil, in humidified air comprised of 20% O_2_, 6% CO_2_ and N_2_ balance. Following maturation, COCs were fixed in 4% paraformaldehyde in phosphate-buffered saline (PBS) and mounted on glass slides using DAKO Fluorescence Mounting Medium (Dako, NSW, Australia). Cu_2_O nanocubes were transferred to water and were drop cast on the biological sample to study the intensity variation between fixed biological sample and Cu_2_O nanocubes.

## Results and Discussion

**Figure 1:**
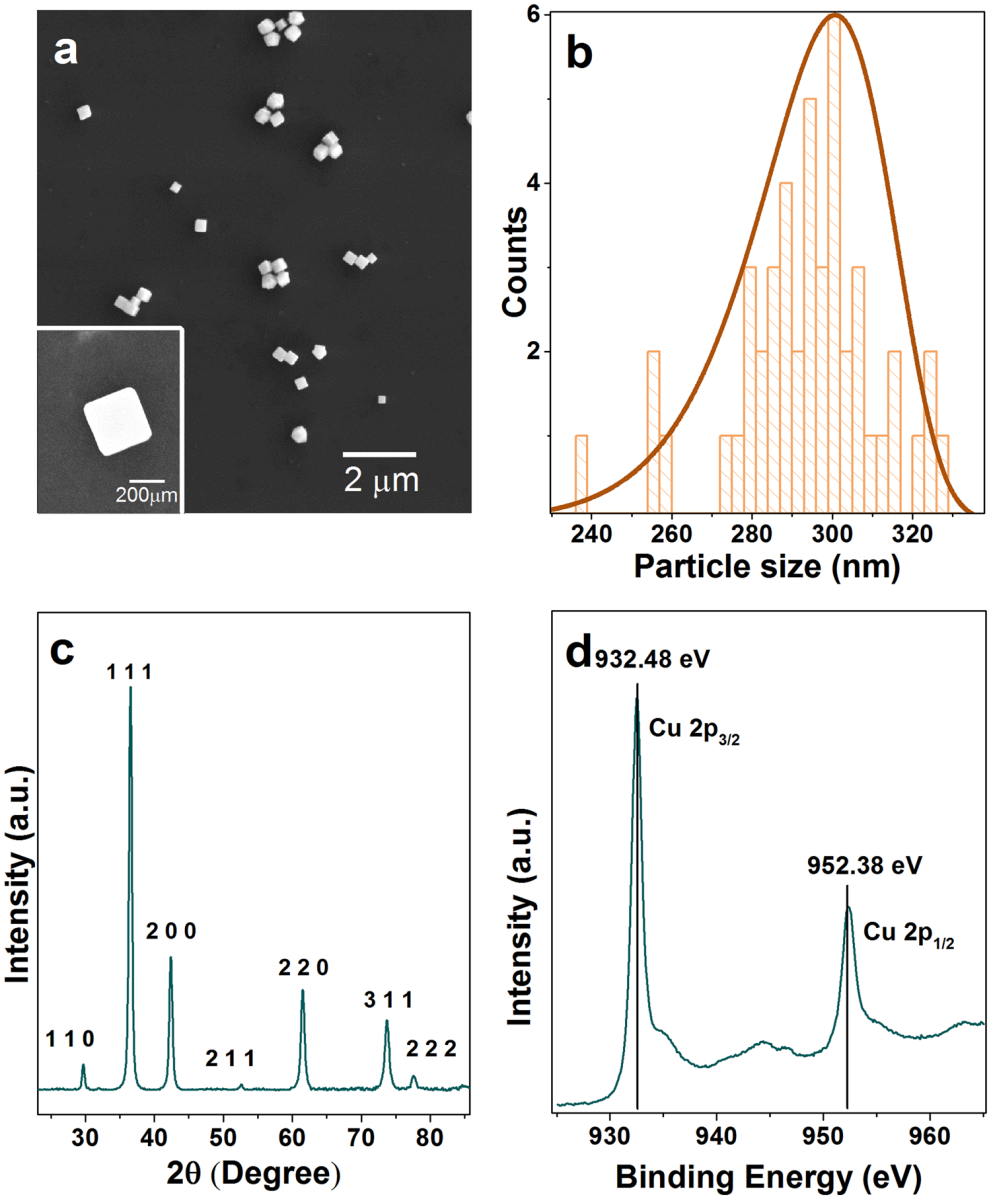
(**a**) SEM image of Cu_2_O nanocubes showing cubic morphology with an individual isolated nanocube shown in the inset. (**b**) Size distribution of each of the Cu_2_O nanocubes analysed with a peak side length of 293 ± 18 nm. (**c**) XRD pattern of Cu_2_O nanocubes where (111) crystal facet has the highest intensity. (**d**) XPS Cu2p scan showing Cu 2p 3/2 peak at 932.5 eV and Cu 2p ½ peak at 952.4 eV with satellite peaks at 943.8 eV and 946.3 eV representing the formation of Cu_2_O with small amounts of the CuO impurities based on the satellite peaks at 944.3 eV and 963.1 eV.

Cu_2_O nanocubes were synthesised using a previously reported seed-mediated approach^1,3^. In this synthesis method, sodium ascorbate acts as a reducing agent, sodium dodecyl sulphate as a capping agent, and sodium hydroxide was used to form Cu(OH)_4_^2−^, which was then reduced to produce Cu_2_O seeds. These seeds produce cubic Cu_2_O nanoparticles after Ostwald ripening and surface reconstruction^1^. SEM images confirmed the truncated cubic shape and smooth surfaces of the particles (Fig. 1a). The average lengths of cubic Cu_2_O are 293 ± 18 nm along one side (Fig. 1b). The X-ray powder diffraction (XRD) pattern of the sample shows the formation of the face-centered cubic lattice Cu_2_O (JCPDF No. 78–2076) (Fig. 1c). To further confirm the formation of Cu_2_O, oxidation state analysis was carried out using X-ray photoelectron spectroscopy (XPS) analysis. The low-resolution XPS survey spectrum (Figure S1) showed the presence of C1s, O1s, Cu2p, and Na1s peak, where C1s is related to the surface adsorbed adventitious carbon while the Na is related to the trace chemicals remaining from the starting materials. It showed that the core level of the Cu 2p3/2 has 932.5 eV and Cu 2p3/2 has 952.4 eV binding energy indicating the oxidation state of Cu(I) (Fig. 1d). Satellite peaks in CuO structures have higher intensities than Cu_2_O structures. Also, the position of these satellite peaks is different in these two oxidation states. The satellite peaks appearing at 943.8 eV and 946.3 eV are related to Cu(I) while the presence of the peaks at 944.3 eV and 963.1 eV relate to the existence of trace CuO impurities^17^. The XRD and XPS results indicate that the synthesised cubic structures are predominantly Cu_2_O structures^3^. Other than the XRD and XPS analysis, the zeta potential of the particles was also measured yielding −17.4 ± 4.7 mV. The zeta potential information is valuable for future functionalizing of the nanocubes with additional materials for targeted biological imaging applications. This result also means that these Cu_2_O nanocubes can increase the surface adsorption ability between nanocubes and charged molecules for biolabelling applications^24^.

**Figure 2:**
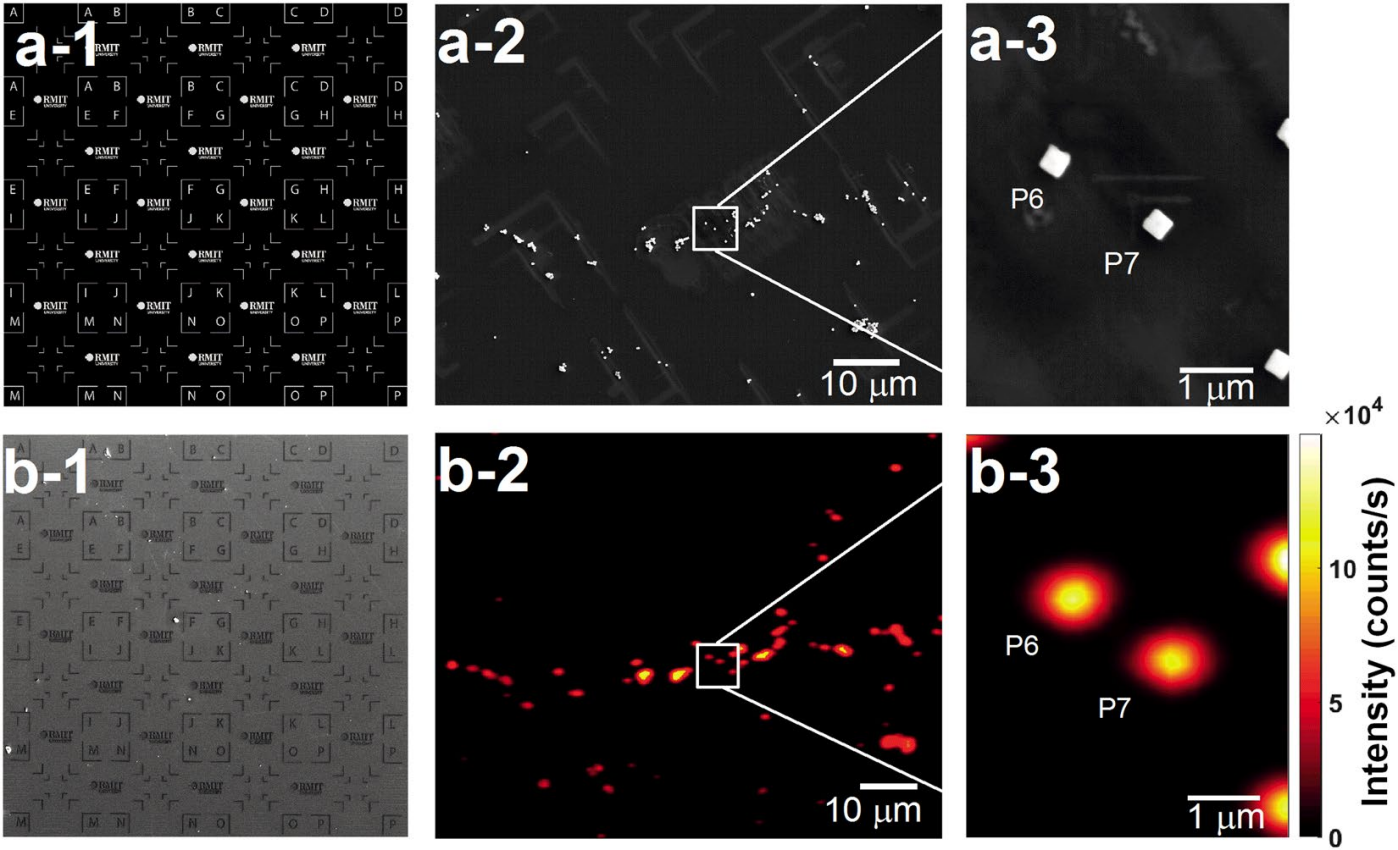
(**a**-**1**) Template of the registration marker which was milled on a silicon substrate using a focused ion beam (FIB) (**b**-**1**). SEM image of Cu_2_O nanocubes dropped cast on a silicon substrate with registration markers to enable the location of the exact area of a certain isolated Cu_2_O nanocube. (**a**-**2**) Low magnification SEM image of Cu_2_O nanocubes. This type of image was taken of different areas of the substrate to select individual nanocubes P1 to p19. (**a-3**) High-resolution SEM image of the boxed region in (**a**-**2**) which was taken after acquiring the optical data to avoid the effect of the electron beam on the optical properties of the Cu_2_O nanocubes. Particles P6 and P7 are shown as an example of two isolated nanocubes. (**b**-**2**) Confocal fluorescence image of the same field-of-view as in (**a**-**2**). (**b**-**3**) High-resolution confocal fluorescence image of the boxed region in (**b**-**2**) of particles P6 and P7.

**Figure 3:**
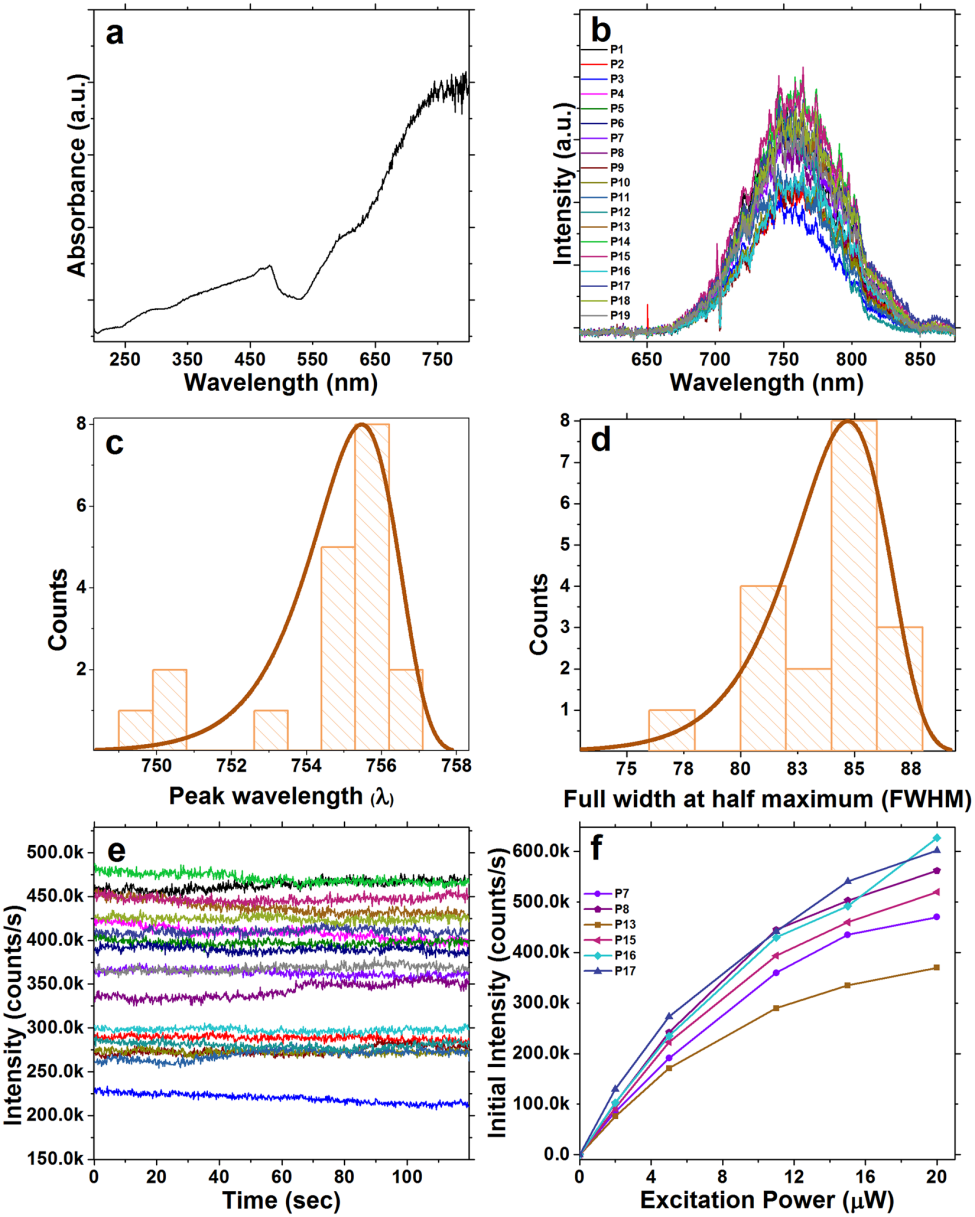
(**a**) UV-visible absorbance spectrum of Cu_2_O nanocubes in water. (**b**) Fluorescence emission spectra of 19 individual Cu_2_O nanocubes excited at 520 nm with a supercontinuum picosecond pulsed laser. (**c**) Emission peak wavelength distribution. The emission peak centered at 754.6 ± 2 nm which can be correlated to oxygen vacancy (V_o_). This wavelength is well suited for biological imaging applications. (**d**) The peak in the distribution of full width at half-maximum (FWHM) emission is around 85 nm. (**e**) Fluorescence intensity of the same nanocubes as in (**a**) over a 120 second time period of continuous excitation with 11 μW time-averaged power at the sample from the supercontinuum pulsed laser. Emission intensities of these individual particles ranged between 226 k to 780 k counts/s. (**f**) Fluorescence emission intensity of 6 selected individual Cu_2_O nanocubes under 2 μW, 5 μW, 11 μW, 15 μW and 20 μW excitation power. The selected nanocubes in (**f**) are a subset, chosen for no particular reason, of those studied in (**b**) and (**e**). This result indicates that individual Cu_2_O nanocubes have the ability to produce considerably bright emission while using low excitation powers.

A template registration marker, shown in Fig 2a-1 was milled into a silicon substrate with a focused ion beam to enable the characterization of isolated Cu_2_O nanocubes. This was followed by drop casting the synthesised nanocubes and drying under air (Fig. 2a-2). A low magnification scanning electron microscope (SEM) image was taken to locate regions of individual and isolated Cu_2_O nanocubes (Figure 2a-2)^23^. The marked silicon platform was used to locate and measure the optical properties of individual particles under a confocal microscope. To confirm the size, morphology, and isolation of particles, high-magnification SEM images were taken after all optical data was acquired from 19 individual nanocubes in order to minimise any effect of possible electron beam damage on their optical properties. Figure 2a-1 represents the template of the registration pattern which has been milled as shown in Fig. 2b-1. A SEM image of the nanocubes on the registered Si wafer is shown in Fig. 2a-2 at low magnification. As an example in Fig 2a-3, we show a high magnification SEM image of two typical individual nanocubes (particles number P6 and P7). Confocal fluorescence images for low and high magnifications are shown in Fig. 2b-2 and b-3, respectively. These images are collected using the same field-of-view as the SEM images shown in Fig. 2a-2 and a-3, respectively, to enable subsequent photostability and spectral measurements.

UV-visible absorbance spectrum of Cu_2_O nanocubes in water was collected, which showed maximum absorbance appeared at 481 nm (Fig. 3a), in addition, the UV-Vis absorbance spectra near-IR absorbance at 750 nm which is well aligned with previously reported literatures^1,3^. Fluorescence spectral data was collected from individual and isolated Cu_2_O particles on the marked silicon substrate using 520 nm (2.38 eV) supercontinuum pulsed laser with 11 µW average excitation power (1.7 W/cm^2^) at room temperature with an FWHM = 10 nm. This pump wavelength was chosen because visible light excitation is compatible with biological imaging^19^. Under these excitation conditions, the emission peak of individual Cu_2_O nanocubes was centred around 754.6 ± 2 nm (Fig. 3b,c) which can be assigned to doubly charged oxygen vacancies (Vo)^18^ in the Cu_2_O nanocube lattice. Having an emission at 754 nm makes this material a promissing candidate for biological imaging as tissue absorption and autofluorescence are minimal in this emission range^19^. The peak in the distribution of full width at half-maximum (FWHM) emission is around 85 nm (Fig. 3d).

In addition to the emission wavelength, brightness and photostability are crucial factors for bioimaging applications. Brightness and photostability data were collected from the same isolated individual Cu_2_O particles (using 520 nm excitation wavelength with 11 µW average power with FWHM = 10 nm for 120 seconds), as shown in Fig. 3e. Emission counts ranged between 226 k and 780 k counts/s, and remained stable for a period of at least 120 seconds, indicating photostable characteristics of the Cu_2_O nanocubes. It should be mentioned that the laser power used in this part of the research is considerably lower than that used for some biological imaging applications^25^. For example, Goetz, M., *et al*. reported confocal imaging during mini-laparoscopy where they used 715 μW average laser power with maximum power limited to 2000 μW^26^. Laser power ranged between 300 mW and 600 mW was used for histomorphologic imaging of brain tumours *in vivo* ^27^.

In addition, the relationship between excitation power and emission counts was studied for 6 individual Cu_2_O particles using 5 excitation powers (Fig. 3f). This study shows that it is possible to observe counts ranging between 76 k counts/s and 130 k counts/s from a single Cu_2_O nanocube using only 2 µW of excitation power with a pulsed laser. The intensity of emitted light was observed to increase with increasing excitation power (Fig. 3f). It is an important factor in bioimaging to use low power excitation lasers as there are reports indicating that higher laser power can damage biological samples^22^. The results showed that when a pulsed laser is used for imaging, saturation has not occurred over a range up to 20 µW laser excitation power. The intensity of the emission from individual Cu_2_O with 20 µW laser excitation was ranged between 470 k counts/s and 602 k counts/s. However, at the higher excitation powers, the intensities of the emission show a non-linear increase and the rate of the increase in the emission counts decreases as shown in supporting information Figure S2 for two individual nanocubes. However, the Cu_2_O nanocubes showed no saturation up to 207 µW average excitation power or 3.16E5 W/cm^2^ excitation power density that means the emission of these nanocubes remains stable even with high laser excitation. The brightness of individual Cu_2_O nanocubes suggests that a lower concentration of this nanomaterial might be required for biological imaging compared to the other fluorophores. Cytotoxicity of Cu_2_O nanoparticles on fish blood has been studied which showed concentration lower than 8 µg/mL has minor toxic effect for living cells^28^. This is an important factor when considering that high concentrations of fluorophores can be toxic to a biological system^29^

**Figure 4:**
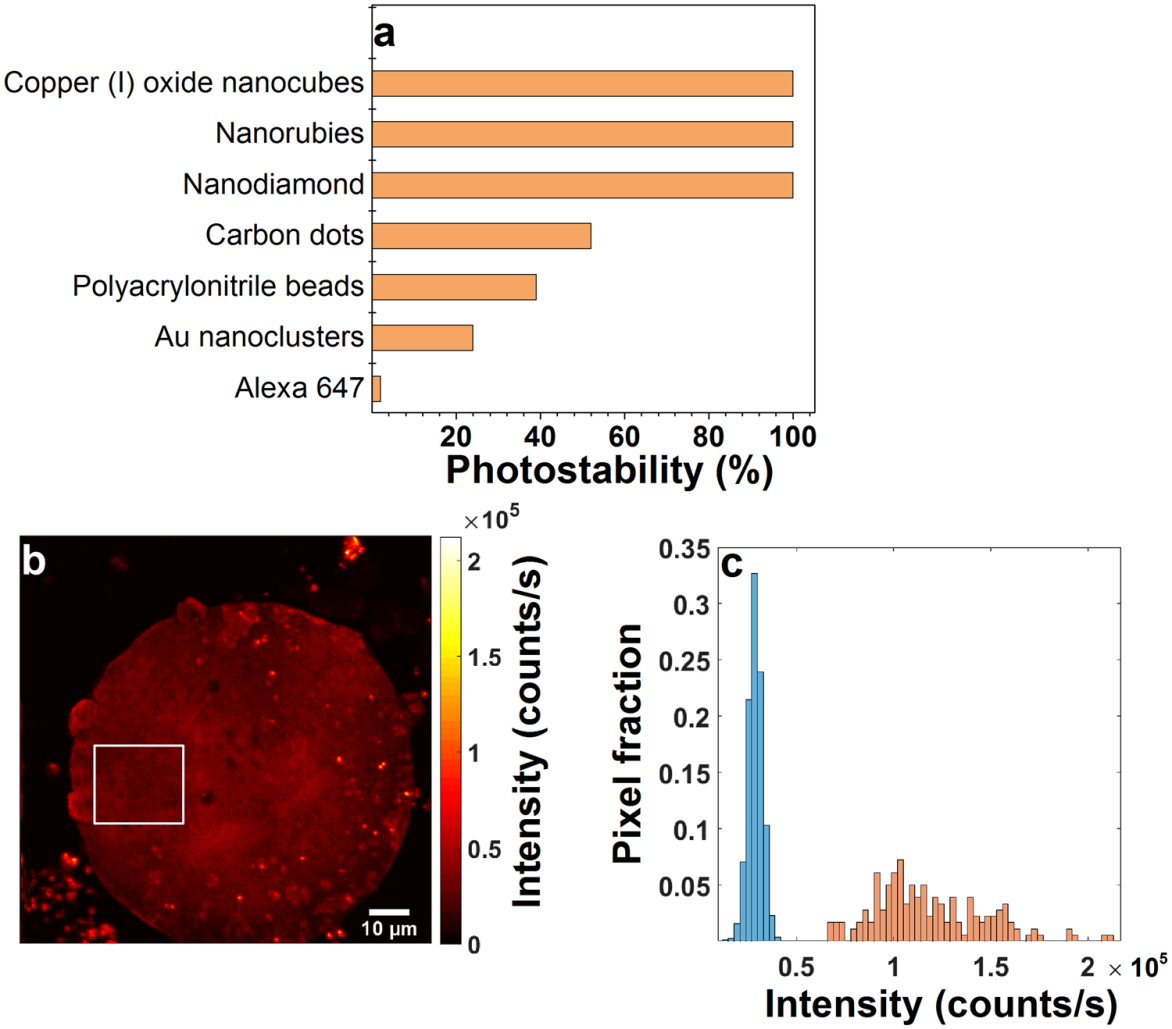
(**a**) Comparison of the photostability of Cu_2_O nanocubes with commercial dyes and emerging fluorescent nanoparticles over a time period of 120 seconds (Table S1)1. The photostability of Cu_2_O nanocubes are 98% higher than the widely used commercial Alexa 647 dye and 48% higher than carbon dots. Cu_2_O nanocubes, nanorubies and nanodiamonds are showing 100% photostability over this time period. (**b**) Confocal fluorescence image of a mouse cumulus-oocyte complex with Cu_2_O nanocubes. Bright dots are Cu_2_O nanocubes. (**c**) Histogram showing the relative brightness of autofluorescence (blue bars) and Cu_2_O nanocube fluorescence (orange bars). The autofluorescence histogram shows the distribution of pixel intensities within the boxed region in (**b**). The Cu_2_O histogram shows the brightness distribution of 20 manually selected Cu_2_O particles in (**b**). The Cu_2_O nanocubes were observed to be approximately 4 times brighter than autofluorescence from the mouse cumulus-oocyte complex hence making them easily distinguishable.

Photostability of the Cu_2_O nanocubes is also remarkable compared to standard commercially available fluorescent probes. It has previously been reported that commercially available fluorescent probes such as Alexa Fluor 647, polyacrylonitrile beads and carbon dots have a short bleaching time which limits their application for long term bioimaging studies during the course of an experiment^21^. Photostability of Cu_2_O was compared with photostability of Alexa 647, polyacrylonitrile beads, Au nanoclusters, carbon dots, nanodiamonds and nanorubies for two minutes^21^. Alexa 647 showed lowest photostability (2%) followed by Au nanoclusters (24%) and polyacrylonitrile beads (39%) (Fig. 4a and Table S1). Photostability of Cu_2_O was the highest (100%) which is similar to nanodiamonds and nanorubies compared to the aforementioned fluorescent probes (Fig. 4a). This comparison further highlights the potential this material has for long term biological imaging applications. A bioimaging demonstration of the intrinsically fluorescent Cu_2_O nanocubes was made through their use as fluorophores coupled with a mouse cumulus-oocyte complex. The Cu_2_O nanocubes were imaged with the same experimental conditions used for optical characterization of individual and isolated nanocubes in Fig. 2. The fluorescence emission of the Cu_2_O nanocubes was compared to the autofluorescence within the mouse cumulus-oocyte complex (Fig. 4b,c). The confocal image of the mouse cumulus-oocyte complex with Cu_2_O nanocubes drop-casted on the surface is shown in Fig. 4b. The bright dots on the surface of the mouse cumulus-oocyte complex correspond to the fluorescence emission from Cu_2_O nanocubes. The histogram in Fig. 4c shows the relative brightness of autofluorescence from the mouse cumulus-oocyte complex compared to Cu_2_O nanocube fluorescence. The autofluorescence histogram collected from the distribution of pixel intensities within the boxed region in Fig. 4b and the Cu_2_O histogram (Fig. 4c) shows the brightness distribution of 20 manually selected Cu_2_O particles in Fig. 4b. Fluorescence from Cu_2_O was observed to be four times higher in intensity compared to the autofluorescence signal from the cumulus-oocyte complex which made them easily distinguishable (Figs. 4c). The attractive characteristics of copper (I) oxide nanocubes, such as their long photostability and high brightness, highlight their potential as an alternative to commercially available fluorescent probes for bioimaging applications.

## Conclusions

In conclusion, we have synthesised copper (I) oxide nanocubes via a seed-mediated method. Individual Cu_2_O nanocubes were studied using a marked substrate which was milled with a focused ion beam to locate and collect optical data from 19 individual particles. This study reveals that single Cu_2_O nanocubes can emit light at a rate of up to 487 K counts/s for at least 120 seconds with only 11 µW (1.7 W/cm^2^) laser excitation. Highly bright and photostable intrinsic fluorescence from copper (I) oxide nanocubes at low excitation powers suggest that the nanocubes are suitable for long time bioimaging experiments. Fluorescence from Cu_2_O nanocubes was also observed to be significantly brighter than the auto-fluorescence from a fixed mouse cumulus-oocyte complex and highly photostable compared to commercially available organic fluorescent materials. However, for in vivo applications, there is further research to be undertaken to determine the biocompatibility of copper (I) oxide nanocubes as a function of their concentration in a biological context.

## Electronic supplementary material

Supporting information
