# Supplementary material for: Fluorescence brightness and photostability of individual copper (I) oxide nanocubes
Source: Sci Rep. 2017 Dec 4;7:16905. doi: 10.1038/s41598-017-17295-0 (PMC5715080; doi:10.1038/s41598-017-17295-0)
Supplement: Supplementary file 1 — Supporting information [file 41598_2017_17295_MOESM1_ESM.doc]

**Supporting Information**

**Fluorescence brightness and photostability of individual copper (I) oxide nanocubes**

Nafisa Zohora^1†^, Ahmad Esmaielzadeh Kandjani^2^, Antony Orth^1^, Hannah M. Brown^3^, Mark R. Hutchinson^4^ and Brant C. Gibson^1†^

*^1^ARC Centre of Excellence for Nanoscale BioPhotonics, School of Science, RMIT University, Melbourne, VIC 3001, Australia*

*^2^Centre for Advanced Materials and Industrial Chemistry, School of Science, RMIT University, Melbourne, VIC 3001, Australia*

*^3^ARC Centre of Excellence for Nanoscale BioPhotonics, Robinson Research Institute, Adelaide Medical School,The University of Adelaide, Adelaide, SA 5005, Australia.*

*^4^ARC Centre of Excellence for Nanoscale BioPhotonics, School of Medicine, University of Adelaide, Adelaide, SA 5005, Australia*

^†^E-mail: nafisa.zohora@rmit.edu.au, brant.gibson@rmit.edu.au, Phone: +61 3 9925 3649, Fax: +61 3 9925 2356.


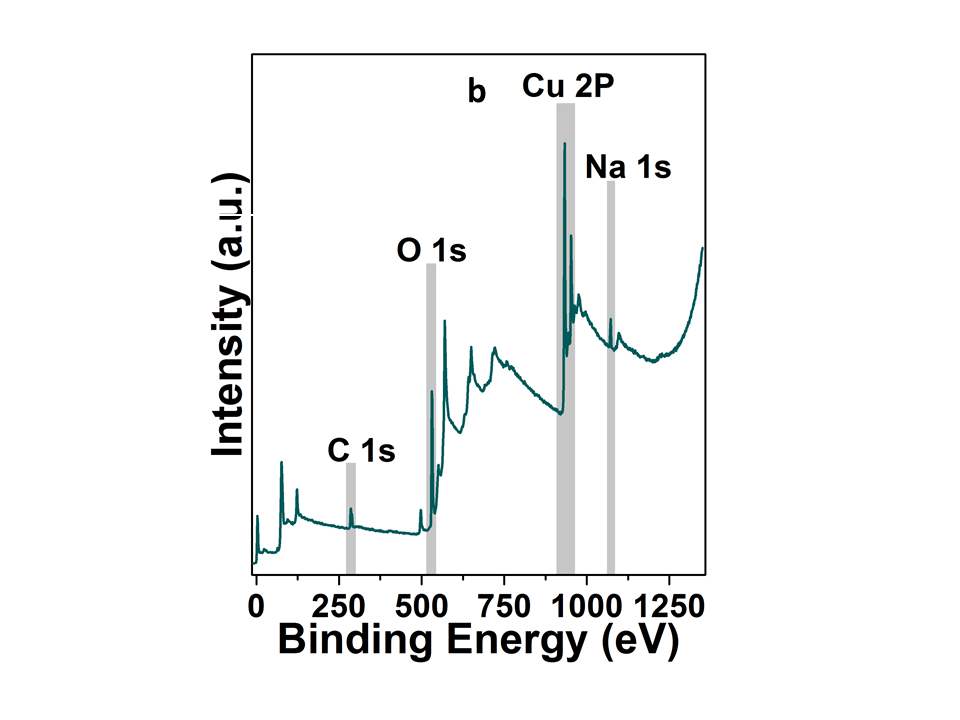


Figure S1:  **XPS survey spectra of Cu2O nanocubes showing the presence of C, O, Cu and Na molecules on the surface as impurities that have been used to synthesise the nanocubes.**


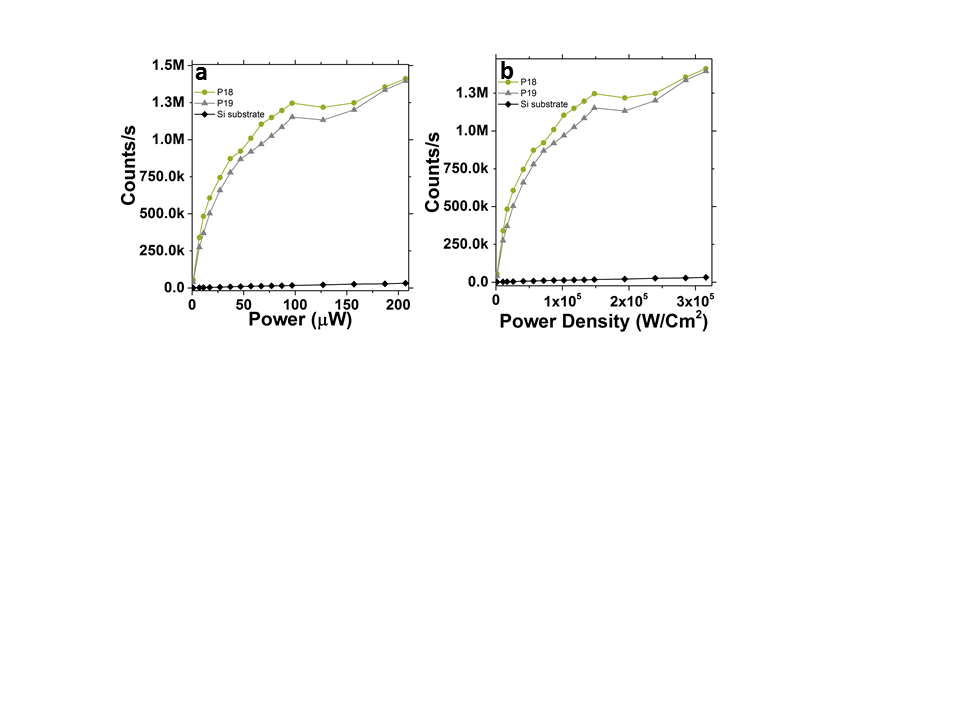


Figure S2: a) Fluorescence emission intensities of two individual Cu2O nanocubes (P18 and P19) as a function of increasing average excitation power. b) Theoretically calculated power density for corresponding average excitation power. Negligible saturation was observed for the individual and isolated Cu2O nanocubes up to (a) 207 µW average excitation power or (b) 3.16E5 W/cm2 excitation power density

Table S1: Comparison of the photostability of Cu_2_O with commercially available dyes and nanoparticles used for biological imaging. [1]

|  | Excitation/emission wavelengths used in this study [nm] | Approximate concentration [µg mL^−1^] | Excitation power  (Wcm^-2^) | Photostability % in 2 minutes |
| --- | --- | --- | --- | --- |
| Alexa 647 | 640/692 | 0.01 (≈8 × 10^−9^ M) | 10.7 | 2 |
| Polyacrylonitrile beads | 640/692 | 4 | 10.7 | 24 |
| Au nanoclusters | 560/692 | 160 | 10.7 | 39 |
| Carbon dots | 470/692 | 60 | 10.7 | 52 |
| Nanodiamonds (NV) | 560/692 | 40 | 10.7 | 100 |
| Nanorubies | 560/692 | 200 | 10.7 | 100 |
| Cu_2_O Nanocubes | 520/758 | One individual dried particle | 3.025 × 10^4^ | 100 |
